# A New Type of Allodiploid Hybrids Derived From Female *Megalobrama amblycephala* × Male *Gobiocypris rarus*

**DOI:** 10.3389/fgene.2021.685914

**Published:** 2021-07-19

**Authors:** Qingfeng Liu, Xuanyi Zhang, Junmei Liu, Fanglei Liu, Fangming Shi, Qinbo Qin, Min Tao, Chenchen Tang, Shaojun Liu

**Affiliations:** ^1^State Key Laboratory of Developmental Biology of Freshwater Fish, Hunan Normal University, Changsha, China; ^2^College of Life Sciences, Hunan Normal University, Changsha, China

**Keywords:** distant hybridization, genetic characteristic, odd number chromosomes, hybrid traits, 5S rDNA

## Abstract

Distant hybridization can combine whole genomes from parent species and result in changes in the phenotypes and genotypes in hybrids. The characteristics of many hybrid fishes with even number of chromosomes have been reported, but the hybrids with odd number chromosomes are rarely reported. Blunt snout bream (*Megalobrama amblycephala*, BSB, 2n = 48) and rare gudgeon (*Gobiocypris rarus*, RG, 2n = 50) belong to two different subfamilies and have quite different biological characteristics. In this study, we obtain the hybrids (BR) derived from the inter-subfamily hybridization of female BSB and male RG. We investigate the fertilization rate, hatching rate, morphological traits, chromosomal numbers, DNA content, growth rates, and 5S rDNA in the BR. The results show that the BR is an allodiploid fish with 49 chromosomes, and all the measurable traits are significantly different (*p* < 0.05) among BR, BSB, and BR. Interestingly, the upper part of the BR body color is similar to BSB (gray), the lower part of the BR body color is similar to RG (light yellow), and the BR inherits a unique light yellow wide longitudinal band from the RG. Furthermore, the BR has a fast growth rate compared with RG. The 5S rDNA of the BR inherits the specific bands of its parental 5S rDNA respectively and has some mutations, which show obvious recombination, heredity, and variability in BR. This study will be of great significance in fish genetic breeding.

## Introduction

Distant hybridization is an important technique and widely used in fish genetic breeding, which can combine whole genomes from parent species and lead to changes in both phenotypes and genotypes in the offspring (Liu, 2010; Zhang et al., 2014; Xu et al., 2015a). For example, in terms of phenotypes, the allodiploid lineage with hybrid traits, high survival rate, good meat quality, and strong stress resistance were established by the distant hybridization of Japanese white crucian carp (*Carassius cuvieri*, ♀) and red crucian carp (*Carassius auratus* red var., ♂) (Wang et al., 2015; Liu et al., 2017, 2018, 2019a,b); the triploid fish with fast growth were produced by the crossing of diploid fish with allotetraploid fish (Tiwary et al., 2004; Zhong et al., 2012; Wang et al., 2020); in terms of genotypes, a high proportion of chimeric genes (>9%) have been found among orthologous genes in the transcriptomes of allotetraploid fish (Liu et al., 2016); many different expressed genes associated with sperm flagellar assembly and motility were found in testis transcriptomes from diploid fish and triploid fish (Xu et al., 2015b; Li et al., 2018a). These phenotypes and genotypes are very important for breeding applications and scientific research. However, these studies on the genetic characteristics of hybrids mostly focus on offspring with even number of chromosomes; there were few reports about offspring with odd number of chromosomes, and hybrids generated from parents with distinct biological characteristics were reported.

Blunt snout bream (*Megalobrama amblycephala*, BSB) and rare gudgeon (*Gobiocypris rarus*, RG) belong to different subfamilies and have quite different biological characteristics. In the catalog, the BSB with 48 chromosomes belongs to *cultrinae*, Cyprinidae, and the RG with 50 chromosomes belongs to *gobioninae*, Cyprinidae^1^. So, the hybridization between the two fish is inter-subfamily hybridization. BSB is characterized by gray body color, spindle with high and thin body, big body (the body length of adult fish is 20–30 cm), 2 years of sexual maturity, and 1 year reproduction cycle (Xiao et al., 2014; Gong et al., 2019). RG is characterized by light yellow body color, spindle body, small body (the body length of adult fish is 3.8–4.5 cm), 4 months of sexual maturity, and breeding all year round (Hong et al., 2016; Wang and Cao, 2017). So, by using BSB and RG as parents for distant hybridization, not only is it possible to obtain hybrid offspring with odd chromosomes, but it also may obtain the offspring with obvious hybrid traits.

In this study, we obtained the hybrid fish (BR) by the distant hybridization derived from female BSB and male RG, which had an odd number of chromosomes and exhibited obvious hybrid traits. Through the research of genetic characteristics of the BR, including morphological traits, ploidy, fertilization rate, hatching rate, growth rates, and 5S rDNA, we aimed to provide an ideal model for the studies of distant hybridization.

## Materials and Methods

### Ethics Statement

The guidelines established by the Administration of Affairs Concerning Animal Experimentation state that approval from the Science and Technology Bureau of China and the Department of Wildlife Administration is not necessary when the fish in question are neither rare nor near extinction (first- or second-class state protection level). Therefore, approval was not required for the experiments described in this manuscript. The fish were deeply anesthetized with 100 mg/L MS-222 (Sigma-Aldrich, St. Louis, MO, United States) prior to dissection. Fish care and experimenters were certified under a professional training course for laboratory animal practitioners held by the Institute of Experimental Animals, Hunan Province, China.

### Production of Hybrids From BSB (♀) × RG (♂)

Individuals of BSB and RG were obtained from the Engineering Research Center of Polyploid Fish Breeding and Reproduction of the State Education Ministry, China, located at Hunan Normal University. During the reproductive season (from May to July) in 2018, mature BSB and RG were chosen as maternal and paternal parents. The mature eggs were obtained by squeezing the belly of BSB, the mature sperm were produced by squeezing the belly of RG, and sperm were collected by capillary glass tubes. The hybrids were formed by fertilization of haploid BSB eggs with haploid RG sperm. The embryos developed in the culture dishes at a freshwater temperature of 22–24°C. The hatched fry of hybrids were transferred to a pond for further culture. Approximately 3,000 embryos were taken at random from the hybrids for the examination of the fertilization rate (number of embryos at the stage of gastrula/total number of eggs taken for fertilization × 100%) and hatching rate (number of hatched fry/number of fertilized eggs × 100%).

### Measurement of Morphological Traits

We randomly selected 30 1-year-old fish (total = 90) from BSB, RG, and BR for morphological examination. The countable traits include the numbers of scales in lateral lines, upper lateral lines, and lower lateral lines, and the numbers of rays in dorsal fins, abdominal fins, and anal fins. The measurable traits include the average values of the whole length (WL), body length (BL), head length (HL), caudal peduncle length (CPL), body height (BH), head height (HH), and caudal peduncle height (CPH). Additionally, we translated the data of measurable traits into proportional data to more clearly show the morphological characteristics. A total of 200 BSB and 200 BR were selected and reared in a 500-m^2^ pond. After feeding for 4 months, body weights (BWs) of BSB and BR were measured for 1 year (*n* = 50 for each fish). Because RG is a kind of small fish, we selected 10 RG (1 year old) to measure their BWs.

### Examination of the Ploidy Level

The DNA content and the chromosome number were used to test the ploidy level of the hybrid offspring. The DNA content of the erythrocytes of BSB, RG, and BR were measured using a flow (Cell Counter Analyzer, Partec, Germany), and in each group, 50 fish (1 year old) were sampled. The blood samples were treated following the method described in the published paper (Liu et al., 2007). The DNA content of each sample was measured under identical conditions. The DNA contents of BSB and RG were used as the controls. Finally, we used a χ^2^-test with Yate’s correction to test for deviation in the ratios of the DNA content of the hybrid offspring to the sum of that from BSB and RG from the expected ratio. The chromosome number was measured using kidney tissues from BR at 10 months of age, respectively, and each sample included 10 fish. The preparations were made according to the method described by Liu et al. (2007) with minor modifications. After culturing for 2–3 days at 20–22°C, the samples were injected 1–3 times with concanavalin at a dose of 6–15 μg/g body weight at an interval of 12–24 h. Two to three hours prior to dissection, each sample was injected with colchicine at a dose of 4–6 μg/g body weight. The kidney tissue was ground in 0.9% NaCl and subjected to hypotonic treatment with 0.075 M KCl at 37°C for 40–60 min and then fixed three times in 3:1 methanol–acetic acid. The cells were added dropwise to cold, wet slides and stained with 4% Giemsa for 40–60 min. The chromosome shape and numbers were analyzed under a light microscope. For each fish sample, 100 metaphase spreads (10 spreads per sample) were examined.

### Genomic DNA Extraction, PCR, and Sequencing

Total genomic DNA was extracted from the peripheral blood cells of BSB, RG, and BR (the age of each fish is 1 year old) using the Universal Genomic DNA Extraction kit (TaKaRa, Dalian, China). Two sets of primers (5S rDNA F: 5′-GCTAT GCCCGATCTCGTCTGA-3′ and R: 5′-CAGGTTGGTATGGCCGTAAGC-3′) were designed by Primers 5 and synthesized to amplify the 5S rDNA from genomic DNA. The PCR reactions were performed in a volume of 25 μl with approximately 20 ng of genomic DNA, 1.5 mM MgCl_2_, 250 μM of each dNTP, 0.4 μM of each primer, and 1.25 U of Taq polymerase (TaKaRa, Dalian, China). The thermal program consisted of an initial denaturation step at 94°C for 5 min followed by 35 cycles (94°C for 35 s, 59°C for 35 s, and 72°C for 35 s) and a final extension step at 72°C for 5 min. The amplification products were separated on a 1.2% agarose gel using TBE buffer. After electrophoresis, the DNA fragments were purified using a gel extraction kit (Sangon, Shanghai, China) and ligated to the pMD18-T vector. The plasmids were transformed into *Escherichia coli* DH5α, and the DNA fragments inserted into the pMD18-T vector were sequenced using an automated DNA sequencer (ABI PRISM 3730, Applied Biosystems, CA, United States). The sequence homology and variation among the fragments amplified from BSB, RG, and BR were analyzed using BioEdit and Clustal W.

## Results

### Fertilization Rate and Hatching Rate of the BR

The results of fertilization rate and hatching rate in BR are shown in Supplementary Table 1 in Supporting Information. We observed 70.3% fertilization rate and 62.6% hatching rate in the hybridization of BSB and RG.

### The Morphological Traits and the Body Weights of the BR

**FIGURE 1:**
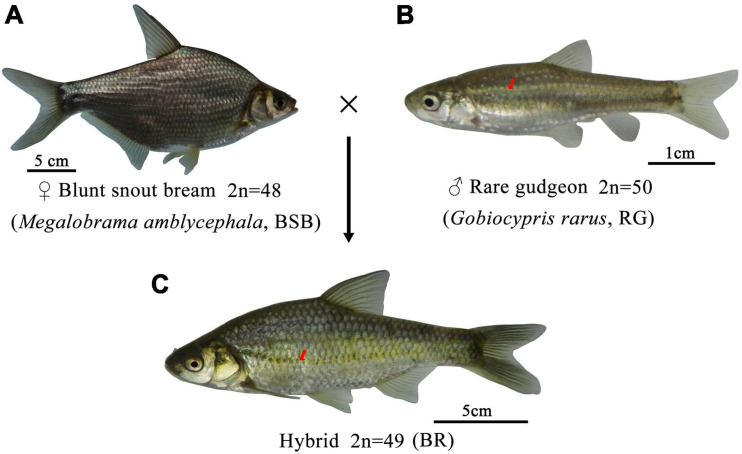
The crossing procedure and the phenotypes of BR and its parents. **(A)** The appearance of BSB, **(B)** the appearance of RG, and **(C)** the appearance of the BR.

The crossing procedure and the phenotypes of BSB, RG, and BR are shown in Figure 1. We observed that the body color of BSB and RG was gray and light yellow, respectively. Interestingly, the color of the upper part of the BR body was gray, the color of the lower part of the BR body was light yellow, and the BR inherited unique light yellow wide longitudinal band from the RG (the red arrow in Figure 1). The counts for lateral line scales, upper lateral line scales, lower lateral line scales, dorsal fins, abdominal fins, and anal fins in BSB, RG, and BR are shown in Table 1. The lateral line scale count is 49–52 (BSB) and 39–40 (BR); the upper lateral line scale count is 9–10 (BSB), 4–5 (RG), and 7 (BR); the lower lateral line scale count is 9–11 (BSB), 5 (RG), and 5 (BR); the dorsal fin count is III + 8–9 (BSB), III + 7–8 (RG), and III + 8–9 (BR); the abdominal fin count is 8–10 (BSB), 8–9 (RG), and 7–8 (BR); the anal fin count is III + 25–27 (BSB), III + 6–7 (RG), and III + 10–12 (BR). The ratios of WL/BL, BL/BH, BL/HL, HL/HH, and CPL/CPH are indicated in Table 2. The ratio of WL/BL is 1.18 ± 0.02 (BSB), 1.27 ± 0.01 (RG), and 1.24 ± 0.02 (BR); the ratio of BL/BH is 2.36 ± 0.12 (BSB), 3.79 ± 0.13 (RG), and 3.26 ± 0.14 (BR); the ratio of BL/HL is 4.61 ± 0.23 (BSB), 4.73 ± 0.25 (RG), and 4.68 ± 0.30 (BR); the ratio of HL/HH is 1.19 ± 0.03 (BSB), 0.96 ± 0.06 (RG), and 1.07 ± 0.07 (BR; the ratio of CPL/CPH is 1.04 ± 0.05 (BSB), 1.69 ± 0.08 (RG), and 1.53 ± 0.03 (BR). In terms of lateral line scales, upper lateral line scales, and anal fins, there were significant differences (*p* < 0.05) among BSB, RG, and BR. In terms of lower lateral line scales, there was no significant difference between RG and BR, but RG and BR were significantly different (*p* < 0.05) from BSB. There were significant differences (*p* < 0.05) among BSB, RG, and BR for all measurable traits. In general, these results indicated that BR showed obvious hybrid traits compare with its parents. The average body weight of 1-year-old BR was 316 g (Table 3), which was significantly higher than that of RG (5.5 g) and was significantly lower than that of BSB (416 g).

**TABLE 1:** Morphological assessment of BSB, RG, and BR.

**Fish type**	**Lateral line scales**	**Upper lateral line scales**	**Lower lateral line scales**	**Dorsal fins**	**Abdominal fins**	**Anal fins**
BSB	49–52^bc^	9–10^bc^	9–11^bc^	III + 8–9	8–10	III + 25–27^bc^
RG	/	4–5^ac^	5^a^	III + 7–8	8–9	III + 6–7^ac^
BR	39–40^ab^	7^ab^	5^a^	III + 8–9	7–8	III + 10–12^ab^

*^a^Represents that it is significantly different from BSB.*

*^b^Represents that it is significantly different from RG.*

*^c^Represents that it is significantly different from BR (p < 0.05).*

**TABLE 2:** The ratios of measurable traits of BSB, RG, and BR.

**Fish type**	**WL/BL**	**BL/BH**	**BL/HL**	**HL/HH**	**CPL/CPH**
BSB	1.18 ± 0.02^bc^	2.36 ± 0.12^bc^	4.61 ± 0.23^bc^	1.19 ± 0.03^bc^	1.04 ± 0.05^bc^
RG	1.27 ± 0.01^a*c*^	3.79 ± 0.13^ac^	4.73 ± 0.25^ac^	0.96 ± 0.06^ac^	1.69 ± 0.08^ac^
BR	1.24 ± 0.02^ab^	3.26 ± 0.14^ab^	4.68 ± 0.30^ab^	1.07 ± 0.07^ab^	1.53 ± 0.03^ab^

*^a^Represents that it is significantly different from BSB.*

*^b^Represents that it is significantly different from RG.*

*^c^Represents that it is significantly different from BR (p < 0.05).*

**TABLE 3:** The average body weight of BSB and BR (g)^a^.

**Fish type**	**4 months**	**6 months**	**8 months**	**10 months**	**12 months**
BSB	63 ± 11*	95 ± 16*	126 ± 17*	283 ± 36*	416 ± 84*
BR	24 ± 7	43 ± 5	61 ± 11	215 ± 11	316 ± 23

^a^Values are mean ± SE (g).

*Represents statistically significant differences between BSB and BR (p < 0.05).

### DNA Content and Chromosome Number of the BR

**FIGURE 2:**
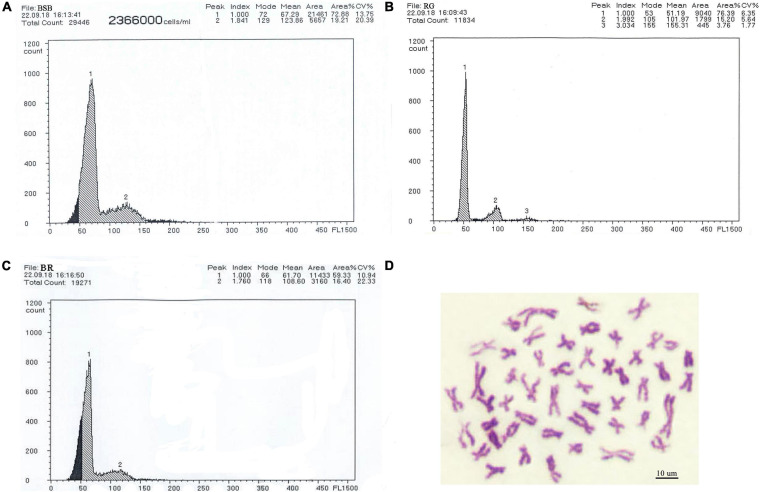
Cytometric histograms of DNA fluorescence and for BSB, RG, and BR, and the metaphase chromosome spreads of BR. **(A)** The mean DNA content of BSB (peak 1:67.29), **(B)** the mean DNA content of RG (peak 1:51.19), **(C)** the mean DNA content of BR (peak 1:61.70), and **(D)** the metaphase chromosome spreads of BR (2n = 49).

The DNA contents of BSB and RG were used as the controls (Figures 2A,B, respectively). The results of the comparison of DNA content between BR and its parents are shown in Supplementary Table 2 in Supporting Information. All the DNA content of the tested fish was equal (*p* > 0.05) to the sum of half of the contents for each of BSB and RG, implying that the BR is an allodiploid fish like its parents (Figure 2C). We tested 100 metaphase spreads of BR, and the chromosome numbers of BR are shown in Supplementary Table 3 in Supporting Information. According to our results, >95% of the tested metaphase spreads had 49 chromosomes, showing that BR is allodiploid fish with 24 chromosomes from BSB and 25 chromosomes from RG (Figure 2D).

### Nucleotide Sequence of 5S rDNA in BR

**FIGURE 3:**
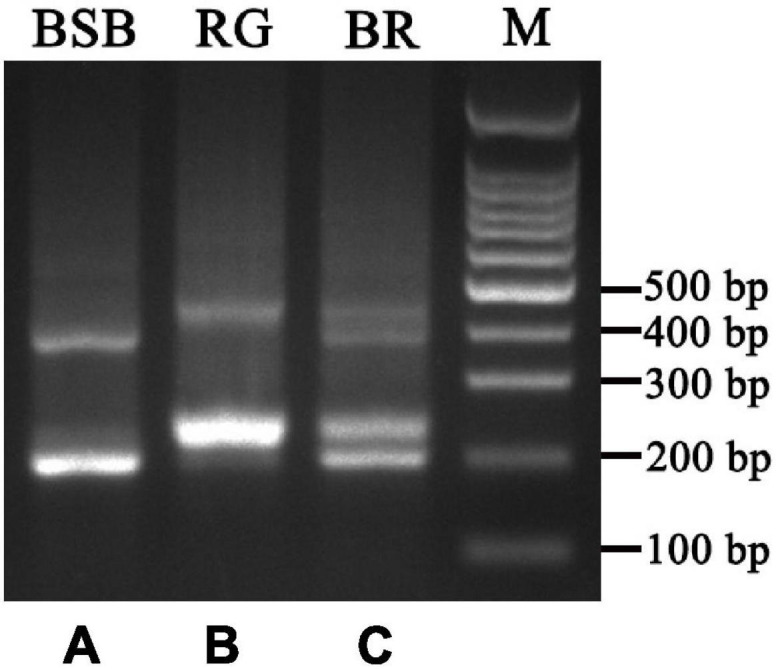
DNA bands amplified from the BSB, RG, and BR. **(A)** Two DNA bands (∼180 and 360 bp) from BSB, **(B)** two DNA bands (∼220 and 440 bp) from 4nAU, and **(C)** four DNA bands (∼180, 220, 360, and 440 bp) from BR.

**FIGURE 4:**
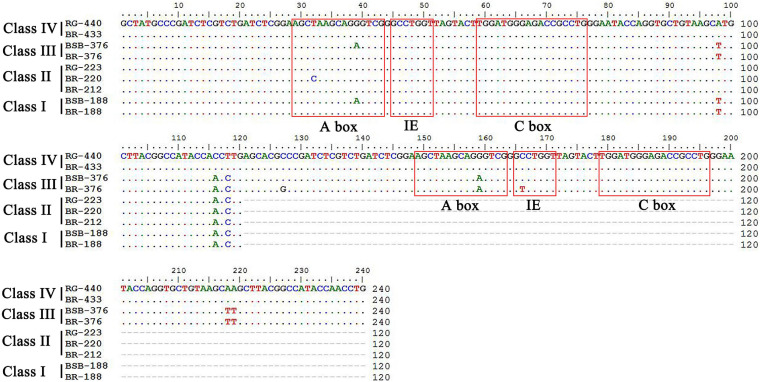
Comparison of the 5S rDNA coding regions from BR, BSB, and RG. ICRs are included in the boxes.

**FIGURE 5:**
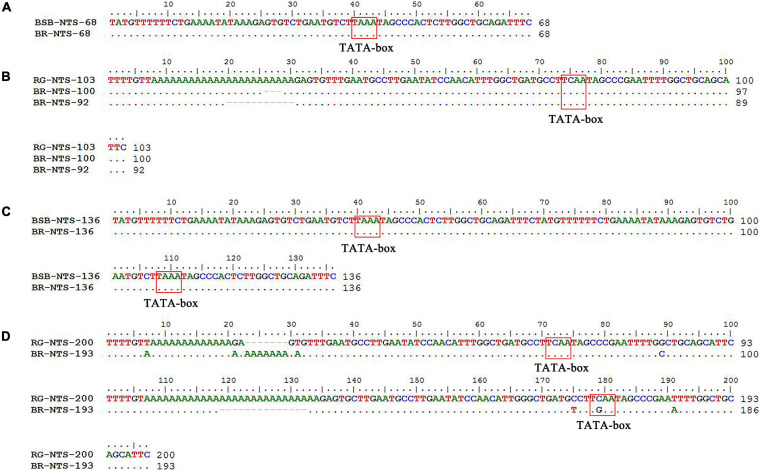
Comparison of the NTS sequences from BR, BSB, and RG **(A–D)**. The NTS upstream TATA-like sequences are included in the boxes.

PCR amplification with 5S primers for BSB, RG, and BR produced distinctive band patterns. There were two bands in BSB, two bands in RG, and four bands in BR (Figure 3). We randomly selected three samples to clone 5S rDNA for each type of fish, and 10 clones were sequenced of every band in each example (a total of 240 clones). The results of Sanger sequencing revealed that the sizes of the two fragments from BSB were 188 and 376 bp, the sizes of the two fragments from RG were 223 and 440 bp, and the sizes of the five fragments from BR were 188, 212, 220, 376, and 433 bp. Using BLASTn, all the fragments were confirmed to be 5S rDNA repeat units, each comprising a 3′ end of the coding region, a whole NTS region, and a large 5′ coding region of the adjacent unit. Nucleotide homology and nucleotide sequence of 5S rDNA among BSB, RG, and BR are shown in Supplementary Tables 4, 5 in Supporting Information. The results showed that the 188 bp of BR was inherited from the 188 bp of BSB (99.47%), the 212 and 220 bp were inherited from the 223 bp of RG (95.07 and 98.21%, respectively), the 376 bp of BR was inherited from the 376 bp of BSB (99.20%), and the 433 bp of BR was inherited from the 440 bp of RG (93.74%). The fragments of 5S rDNA in BR (designated class I: 188 bp, class II: 212 or 220 bp, class III: 376 bp, and class IV: 433 bp) (Figure 4) were categorized into different NTS types (designated NTS-63, NTS-92 or NTS-100, NTS-136, and NTS-193 for 63, 92 or 100, 136, and 193 bp, respectively) (Figure 5). All the internal control regions (including Abox, internal element and C box) were identified in the coding regions (Figure 4), and the TATA box control element, upstream from the next array, was examined within all the NTS sequences, which has been modified to TA(C/G)AA (Figure 5). In short, the coding region sequence data and the NTS sequence data of BR inherited the genetic characteristics from its parents and with some mutant bases and indels (Figures 4, 5).

## Discussion

Distant hybridization is defined as inter-specific or supra-specific hybridization; it is an important technique for preventing variety degeneration and improving variety (Wang et al., 2019). If the progeny derived from distant hybridization are bisexual and fertile, they can form new species or lineages. In previous studies, many kinds of fishes were obtained by distant hybridization, such as the following: in different species, the allodiploid fish lineage (2n = 100) was formed by Japanese white crucian carp (♀) × red crucian carp (♂) (Liu et al., 2019a); in different genera, the allotetraploid fish lineage (4n = 200) was obtained by red crucian carp (♀) × common carp (*Cyprinus carpio*, ♂) (Liu et al., 2001) and the allodiploid fish lineage (2n = 48) was produced by blunt snout bream (♀) × topmouth culter (*Culter alburnus*, ♂) (Xiao et al., 2014; Ren et al., 2019); in different subfamilies, the diploid gynogenetic fish (2n = 48) and triploid hybrid fish (3n = 72) were obtained by grass carp (*Ctenopharyngodon idellus*, ♀) × topmouth culter (♂) (Wu et al., 2018). Through long-term and systematic studies on distant hybridization of fishes, the researchers have reported a lot of studies of genetic and reproductive characteristics in fish distant hybridization (Wang et al., 2019; Gong et al., 2020; Hu et al., 2020; Liu et al., 2020). However, most of these reports were based on the hybrid offspring with even number chromosomes; there are few reports on the genetic characteristics of hybrid offspring with odd number chromosomes. Therefore, a hybrid fish with odd number chromosomes is of great significance for exploring the genetic law of distant hybridization. On the other hand, there were few reports of hybridization between parents with very different traits, especially in terms of individual size and body shape. Fortunately, we successfully obtained the hybrid fish (BR) by the inter-subfamily hybridization of female BSB and male RG.

Distant hybridization is usually accompanied by the appearance of polyploidy, and diploid hybrid progenies are experiencing difficultly surviving in distant hybridization, especially in different subfamilies (Chen et al., 2018). For instance, in different subfamilies, gynogenetic diploid red crucian carp (2n = 100), triploid hybrid fish (3n = 124), and tetraploid hybrid offspring (4n = 148) have been produced by the hybridization of red crucian carp (♀) and blunt snout bream (♂) (Qin et al., 2014); the diploid gynogenetic grass carp (2n = 48) and triploid hybrid fish (3n = 72) were obtained by the hybridization of grass carp (♀) and topmouth culter (♂) (Wu et al., 2018); and triploid hybrid fish (3n = 72) and a small proportion diploid hybrid fish (2n = 48) were produced by the hybridization of grass carp (♀) and blunt snout bream (♂) (He et al., 2013). Interestingly, in this study, all the progenies (BR) were allodiploid hybrid fish with 49 chromosomes (2n = 49); it is a rare phenomenon in distant hybridization between different subfamilies. We guess that this may be because the nucleus–cytoplasm or nucleus–nucleus compatibility between the BSB and the RG is good. When the genetic material between the two parents is not compatible, polyploidy usually tends to be produced (Robertson et al., 2010; Wang et al., 2013). For example, the incompatibilities of the genetic material between red crucian carp and common carp led to the formation of the unreduced gametes by endoreduplication, endomitosis, or the fusion of germ cells in the F_2_ hybrids, and the allotetraploid fish were obtained in F_3_ (Liu, 2010). This result indicates that although the two species have a far relationship, the compatibility between them may be good. Furthermore, the formation of allodiploid BR supported the genetic rules summarized in previous reports: when the number of the maternal chromosomes is equal or similar (no significant difference) to that of the paternal chromosomes, the allodiploid fish can be produced (Liu et al., 2020). Distant hybridization is usually accompanied by changes in phenotypes. According to our results, all the measurable traits and most countable traits are significantly different (*p* < 0.05) among BR, BSB, and RG (Tables 1, 2), and BR showed obvious hybrid characteristics. Interestingly, in terms of body color, the upper part of the BR body is similar to BSB (gray), the lower part of the BR body is similar to RG (light yellow), and the BR inherited unique light yellow wide longitudinal band from the RG (the red arrow in Figure 1). In general, BR had obvious hybrid characteristics in appearance. According to these results, for the first view, we speculated that the production of these obvious traits may be due to the greater genetic distance between the parents and the large difference in biological traits between the parents. For another view, the BR did not look more like RG, even if BR inherited more chromosomes from RG than that from BSB. This may be because there was no significant difference between the number of chromosomes from BSB and the number of chromosomes from RG in the BR. The average body weight of 12-month-old BR was 316 g; it was significantly higher than RG (5.5 g) but lower than BSB (416 g). The faster growth rate of BR is probably affected by genetic material from BSB. In future research, we will use genome sequencing, transcriptome sequencing, gene editing, and other methods to study the molecular mechanism of these hybrid traits.

The genomes of parents can be combined in the distant hybridization, which lead to changes in gene structure and gene expression in the hybrids (Li et al., 2018b). In previous studies, researchers found that there are many chimeric genes and mutant genes in the allopolyploid fish and allodiploid fish by analysis of genome or transcriptome sequencing (Liu et al., 2016, 2018); in many hybrid fishes, the 5S rDNA has also undergone base recombination, which is a good marker for understanding the variability, organization, and evolution of fish species (Martins and Galetti, 2001; Campo et al., 2009; Merlo et al., 2012). According to the results of 5S rDNA, there were two specific bands in BSB (188 and 376 bp) and two specific bands in RG (223 and 440 bp) (Figure 3); interestingly, the BR inherited all the specific bands from both parents, showing the characteristics of recombination, mutation, and deletion (Figures 3–5). This recombination is different from previous reports wherein only the bases between the parents were recombined; the 5S rDNA of BR was formed by recombination of two different gene sequences. We thought that this may be the result of the fusion of the two genomes and the BR contained two subgenomes from the parents. Furthermore, the 5S rDNA of BR is a good molecular marker for identifying hybrids.

In general, we produced a new allodiploid fish with odd number chromosomes, and we showed the hybridity and variability by analyzing the morphological traits, body color, ploidy, and 5S rDNA. The study enriched the genetic characteristics of the hybrid offspring with odd number chromosomes, and the BR provided a good material for the study of hybrid traits in hybrid fish, especially in growth, body type, and body color.

## Data Availability Statement

The original contributions presented in the study are included in the article/Supplementary Material, further inquiries can be directed to the corresponding author/s.

## Ethics Statement

The animal study was reviewed and approved by the Institute of Experimental Animals, Hunan Province, China.

## Author Contributions

QL and SL: conceive and design of this study. QL: carry out most statistical analyses, the writing of the manuscript, primers design, and photograph collection. QL, XZ, and JL: experimental work. FL and FS: experimental material collection. SL, MT, QQ, and CT: manuscript modification. All authors read and approved the final manuscript.

## Conflict of Interest

The authors declare that the research was conducted in the absence of any commercial or financial relationships that could be construed as a potential conflict of interest.

## Footnotes

1 www.fishbase.org
