# Supplementary material for: A New Type of Allodiploid Hybrids Derived From Female Megalobrama amblycephala × Male Gobiocypris rarus
Source: Front Genet. 2021 Jul 19;12:685914. doi: 10.3389/fgene.2021.685914 (PMC8327091; doi:10.3389/fgene.2021.685914)
Supplement: Supplementary file 1 [file Table_1.docx]

Supplementary Information Appendix for:

A new type of allodiploid hybrids derived from female *Megalobrama amblycephala* × male *Gobiocypris Rarus*

Qingfeng Liu^a,b,1^, Xuanyi Zhang^a,b,1^, Junmei Liu^a,b^, Fanglei Liu^a,b^, Fangming Shi^b^, Qinbo Qin^a,b^, Min Tao^a,b^, Chenchen Tang^a,b^, Shaojun Liu ^a,b,*^

^a^ *State Key Laboratory of Developmental Biology of Freshwater Fish, Hunan Normal University, Changsha, 410081, Hunan, P.R. of China;*

^b^ *College of Life Sciences, Hunan Normal University, Changsha, 410081, Hunan, P. R. of China.*

*Correspondence to ShaoJun Liu

E-mail, [lsj@hunnu.edu.cn](mailto:lsj@hunnu.edu.cn),

^1^ These authors contributed equally to this work.

1. SUPPLEMENTARY TABLES

Supplementary Table S1. The fertilization rate and hatching rate of BR.

| Fish type | Fertilization rate | Hatching rate |
| --- | --- | --- |
| BR | 70.3% | 62.6% |

Supplementary Table S2. Mean DNA content of BSB, RG, and BR.

| Species/hybrids | Mean DNA content |  | Ratio |  |
| --- | --- | --- | --- | --- |
|  |  |  | Observed | Expected |
| BSB | 67.29 |  |  |  |
| RG | 51.19 |  |  |  |
| BR | 61.70 |  | BR/(0.5BSB+0.5RG) = 1.04^a^ | 1 |

^a^ The observed ratio is not significantly different (*P* > 0.05) from the expected ratio.

Supplementary Table S3. Chromosome number at mitotic metaphase in BR.

| Fish type | Number of samples | Number of metaphase spreads |  | Chromosome number^a^ | | |  |  | |
| --- | --- | --- | --- | --- | --- | --- | --- | --- | --- |
|  |  |  |  | < 47 | 47-48 | 49 | | |  |
| BR | 10 | 100 |  | 1 | 4 | 95 | | |  |

^a^ Values represent the number of individuals counted with the specified chromosome number.

Supplementary Table S4. Nucleotide homology of 5S rDNA sequences among BSB, RG, and BR (percentage).

| Type of 5S rDNA in BR | Length | BR/BSB | BR/RG |
| --- | --- | --- | --- |
| 5s-188 | 188 bp | 99.47% | / |
| 5s-220 | 220 bp | / | 98.21% |
| 5s-212 | 212 bp | / | 95.07% |
| 5s-376 | 376 bp | 99.20% | / |
| 5s-433 | 433 bp | / | 93.74% |

Supplementary S5. The sequences of 5S rDNA among BSB, RG, and BR.

>BSB-188

GCTATGCCCGATCTCGTCTGATCTCGGAAGCTAAGCAGAGTCGGGCCTGGTTAGTACTTGGATGGGAGACCGCCTGGGAATACCAGGTGCTGTAAGCTTTATGTTTTTTCTGAAAATATAAAGAGTGTCTGAATGTCTTAAATAGCCCACTCTTGGCTGCAGATTTCGCTTACGGCCATACCAACCTG

>BSB-376

GCTATGCCCGATCTCGTCTGATCTCGGAAGCTAAGCAGAGTCGGGCCTGGTTAGTACTTGGATGGGAGACCGCCTGGGAATACCAGGTGCTGTAAGCTTTATGTTTTTTCTGAAAATATAAAGAGTGTCTGAATGTCTTAAATAGCCCACTCTTGGCTGCAGATTTCGCTTACGGCCATACCAACCTGAGCACGCCCGATCTCGTCTGATCTCGGAAGCTAAGCAGAGTCGGGCCTGGTTAGTACTTGGATGGGAGACCGCCTGGGAATACCAGGTGCTGTAAGCTTTATGTTTTTTCTGAAAATATAAAGAGTGTCTGAATGTCTTAAATAGCCCACTCTTGGCTGCAGATTTCGCTTACGGCCATACCAACCTG

>RG-223

GCTATGCCCGATCTCGTCTGATCTCGGAAGCTAAGCAGGGTCGGGCCTGGTTAGTACTTGGATGGGAGACCGCCTGGGAATACCAGGTGCTGTAAGCATTTTTGTTAAAAAAAAAAAAAAAAAAAAAAAGAGTGTTTGAATGCCTTGAATATCCAACATTTGGCTGATGCCTTCAATAGCCCGAATTTTGGCTGCAGCATTCGCTTACGGCCATACCAACCTG

>RG-440

GCTATGCCCGATCTCGTCTGATCTCGGAAGCTAAGCAGGGTCGGGCCTGGTTAGTACTTGGATGGGAGACCGCCTGGGAATACCAGGTGCTGTAAGCATTTTTGTTAAAAAAAAAAAAAGAGTGTTTGAATGCCTTGAATATCCAACATTTGGCTGATGCCTTCAATAGCCCGAATTTTGGCTGCAGCATTCGCTTACGGCCATACCACCTTGAGCACGCCCGATCTCGTCTGATCTCGGAAGCTAAGCAGGGTCGGGCCTGGTTAGTACTTGGATGGGAGACCGCCTGGGAATACCAGGTGCTGTAAGCAATTTTGTAAAAAAAAAAAAAAAAAAAAAAAAAAAAGAGTGCTTGAATGCCTTGAATATCCAACATTGGGCTGATGCCTTCAATAGCCCGAATTTTGGCTGCAGCATTCGCTTACGGCCATACCAACCTG

>BR-188

GCTATGCCCGATCTCGTCTGATCTCGGAAGCTAAGCAGGGTCGGGCCTGGTTAGTACTTGGATGGGAGACCGCCTGGGAATACCAGGTGCTGTAAGCTTTATGTTTTTTCTGAAAATATAAAGAGTGTCTGAATGTCTTAAATAGCCCACTCTTGGCTGCAGATTTCGCTTACGGCCATACCAACCTG

>BR-220

GCTATGCCCGATCTCGTCTGATCTCGGAAGCCAAGCAGGGTCGGGCCTGGTTAGTACTTGGATGGGAGACCGCCTGGGAATACCAGGTGCTGTAAGCATTTTTGTTAAAAAAAAAAAAAAAAAAAAGAGTGTTTGAATGCCTTGAATATCCAACATTTGGCTGATGCCTTCAATAGCCCGAATTTTGGCTGCAGCATTCGCTTACGGCCATACCAACCTG

>BR-212

GCTATGCCCGATCTCGTCTGATCTCGGAAGCTAAGCAGGGTCGGGCCTGGTTAGTACTTGGATGGGAGACCGCCTGGGAATACCAGGTGCTGTAAGCATTTTTGTTAAAAAAAAAAAAGAGTGTTTGAATGCCTTGAATATCCAACATTTGGCTGATGCCTTCAATAGCCCGAATTTTGGCTGCAGCATTCGCTTACGGCCATACCAACCTG

>BR-376

GCTATGCCCGATCTCGTCTGATCTCGGAAGCTAAGCAGGGTCGGGCCTGGTTAGTACTTGGATGGGAGACCGCCTGGGAATACCAGGTGCTGTAAGCTTTATGTTTTTTCTGAAAATATAAAGAGTGTCTGAATGTCTTAAATAGCCCACTCTTGGCTGCAGATTTCGCTTACGGCCATACCAACCTGAGCACGGCCGATCTCGTCTGATCTCGGAAGCTAAGCAGAGTCGGGTCTGGTTAGTACTTGGATGGGAGACCGCCTGGGAATACCAGGTGCTGTAAGCTTTATGTTTTTTCTGAAAATATAAAGAGTGTCTGAATGTCTTAAATAGCCCACTCTTGGCTGCAGATTTCGCTTACGGCCATACCAACCTG

>BR-433

GCTATGCCCGATCTCGTCTGATCTCGGAAGCTAAGCAGGGTCGGGCCTGGTTAGTACTTGGATGGGAGACCGCCTGGGAATACCAGGTGCTGTAAGCATTTTTGTAAAAAAAAAAAAAAAAAAAAAAAGAGTTTGAATGCCTTGAATATCCAACATTTGGCTGATGCCTTCAATAGCCCGAATTTTGCCTGCAGCATTCGCTTACGGCCATACCACCTTGAGCACGCCCGATCTCGTCTGATCTCGGAAGCTAAGCAGGGTCGGGCCTGGTTAGTACTTGGATGGGAGACCGCCTGGGAATACCAGGTGCTGTAAGCAATTTTGTAAAAAAAAAAAAAAGAGTGCTTGAATGCCTTGAATATCCAACATTGGGCTGATGTCTTGAATAGCCCGAAATTTGGCTGCAGCATTCGCTTACGGCCATACCAACCTG
